# A Case of Atypical Meig’s Syndrome Presenting With Pleural and Pericardial Effusion

**DOI:** 10.7759/cureus.77976

**Published:** 2025-01-25

**Authors:** Marwa Mir, Bella A Gnakou, Hamid Shaaban, Gunwant Guron, Zafar Jamil

**Affiliations:** 1 Internal Medicine, Saint Michael's Medical Center, Newark, USA; 2 Internal Medicine, Morristown Medical Center, Morristown, USA; 3 Hematology and Oncology, Saint Michael's Medical Center, Newark, USA; 4 Hematology and Oncology, New York Medical College, Newark, USA; 5 Vascular Surgery, Saint Michael's Medical Center, Newark, USA

**Keywords:** ascites, meig syndrome, ovarian tumours, pericardial effusion, rare cause of pleural effusion

## Abstract

Meigs syndrome is a rare condition characterized by the triad of benign ovarian tumors, ascites, and pleural effusion. However, concurrent presentation with both pleural and pericardial effusions is exceedingly rare. This paper presents a unique case of Meigs syndrome in a 75-year-old African American female patient with a history of anemia who was admitted for a left femur fracture secondary to a fall.

In addition to her orthopedic injury, the patient exhibited intermittent abdominal fullness and discomfort, prompting a thorough diagnostic evaluation. Physical examination was unremarkable, and laboratory investigations revealed microcytic anemia with normal renal and hepatic function tests. Unexpectedly, imaging studies revealed the presence of a large lobulated enhancing mass lesion arising from the terminal ileum, with no evidence of ascites. Computed tomography (CT) imaging also revealed bilateral pleural effusions and a mild pericardial effusion. Initial diagnostic evaluations yielded no significant findings with normal CA-125 and CEA levels and no abnormalities in peripheral blood.

The pathophysiology underlying the development of pleural and pericardial effusions in Meigs syndrome remains unclear but is thought to involve lymphatic obstruction and increased vascular permeability secondary to the ovarian tumor.

In conclusion, this case underscores the importance of recognizing variant presentations of Meigs syndrome, particularly in the context of concurrent pleural and pericardial effusions, which may pose diagnostic challenges but warrant prompt identification and management to optimize patient outcomes.

## Introduction

Meigs syndrome, a rare clinical entity characterized by a triad of benign ovarian fibroma, ascites, and pleural effusion, was first delineated by Meig in 1937 [1]. Conventionally, Meigs syndrome has been associated with ovarian tumors, particularly fibromas, leading to the accumulation of ascitic fluid and pleural effusions. This syndrome represents a diagnostic challenge due to its varied presentation and association with other conditions mimicking its features. While the classical triad is well-documented, atypical presentations of Meigs syndrome, such as the concurrent occurrence of pleural and pericardial effusions, remain exceedingly rare and poorly understood. To date, only a limited number of cases have been reported in the medical literature, highlighting the paucity of data on this atypical presentation [2, 3].

The syndrome's classical presentation predominantly occurs in postmenopausal women, with ovarian fibromas representing the primary tumor type implicated in its pathogenesis [4]. Pleural effusions, a common manifestation of Meigs syndrome, have been extensively documented in the medical literature. Ovarian fibromas, though benign, can cause fluid accumulation in the peritoneal cavity, leading to ascites, and may also stimulate the secretion of pleural effusions. These effusions are typically unilateral and resolve promptly after surgical removal of the ovarian tumor, distinguishing them from malignant processes [5, 6]. 

While pleural effusions are a well-recognized component of Meigs syndrome, concurrent pericardial effusion in this context is an exceedingly rare occurrence. The underlying mechanisms contributing to the development of pericardial effusions in Meigs syndrome remain poorly understood, warranting further investigation. 

Herein, we present a compelling case of a 75-year-old African American female patient presenting with a rare phenomenon of concurrent pleural and pericardial effusions in Meigs syndrome with no ascites. 

## Case presentation

A 75-year-old African American female patient with a history of hypertension, peripheral arterial disease, and hyperlipidemia presented to the hospital following a fall, resulting in a left femoral neck fracture. In addition to the injury, she reported intermittent abdominal fullness and discomfort that had been occurring for the past few months. She denied nausea or vomiting. The patient also noted a gradual onset of worsening shortness of breath (SOB), prompting further investigation.

**Figure 1:**
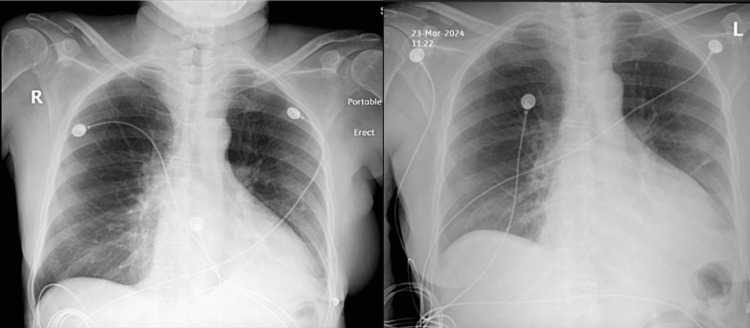
The chest X-ray shows mild bilateral pleural effusion, greater on the left than the right, with moderate collapse/consolidation of the basal segments of the left lower lobe.

On physical examination, the patient’s cardiopulmonary exam was unremarkable, with normal heart sounds and clear lungs. However, imaging studies revealed mild cardiomegaly on chest X-ray and a small left-sided pleural effusion, which prompted additional evaluation (Figure 1). Laboratory tests showed severe microcytic anemia with thrombocytopenia, consistent with iron deficiency anemia (hemoglobin: 6.3 g/dL), for which she received a transfusion of three units of packed red blood cells. Thyroid function tests indicated probable subclinical hypothyroidism (thyroid-stimulating hormone (TSH): 0.203 mIU/L with free thyroxine (T4): 1.48 ng/dL).

**Figure 2:**
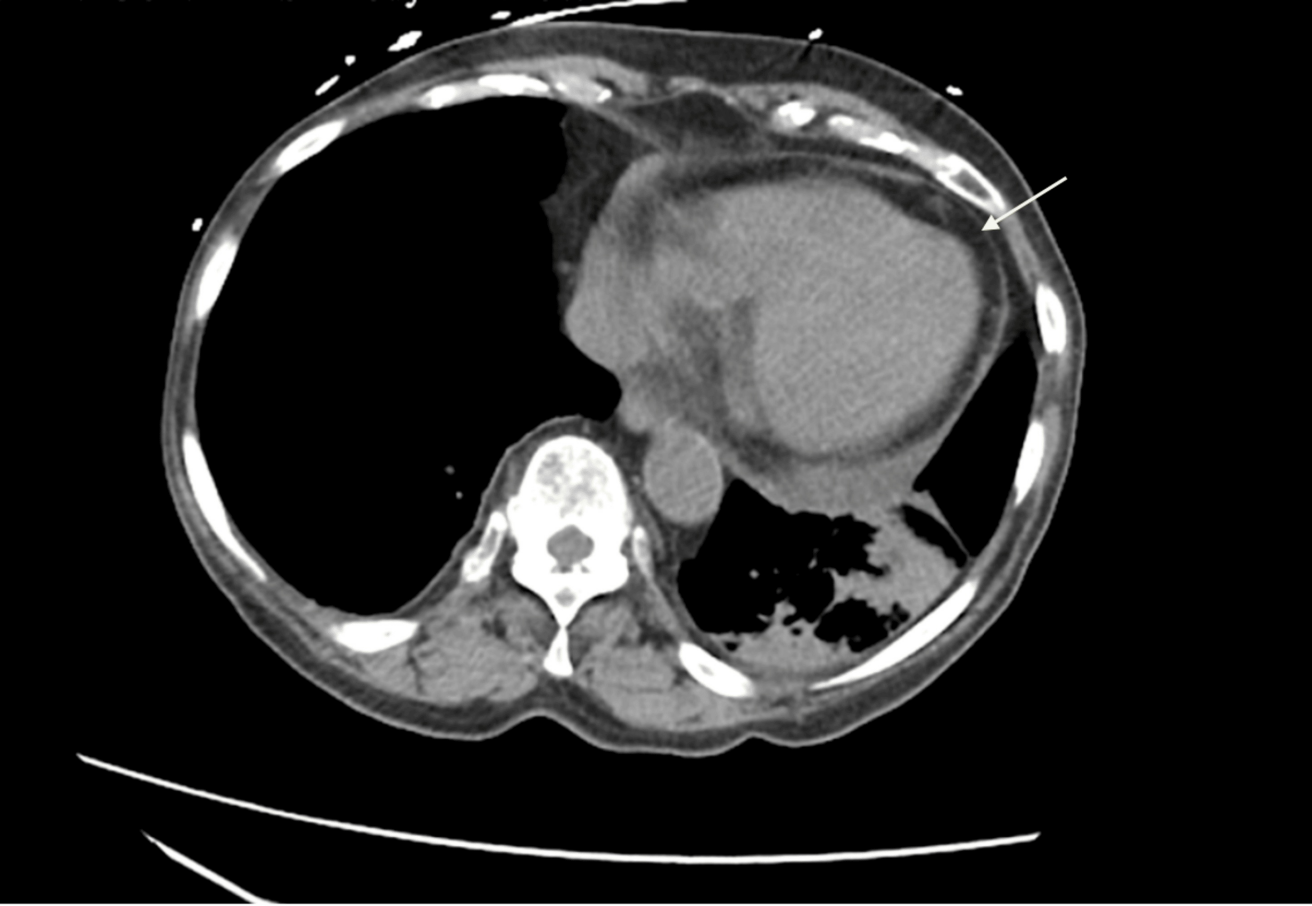
Axial view of the CT of the abdomen and pelvis without contrast shows pericardial effusion (maximum thickness of 1.5 cm along the left ventricle).

Computed tomography (CT) imaging of the abdomen and pelvis revealed important findings: mild bilateral pleural effusions (more prominent on the left), moderate collapse or consolidation of the basal segments of the left lower lobe, and volume loss in the left hemithorax. A mild pericardial effusion was also noted, with a maximum thickness of 1.5 cm along the left ventricle, raising concern for pericardial involvement from an inflammatory or malignant process (Figure 2).

**Figure 3:**
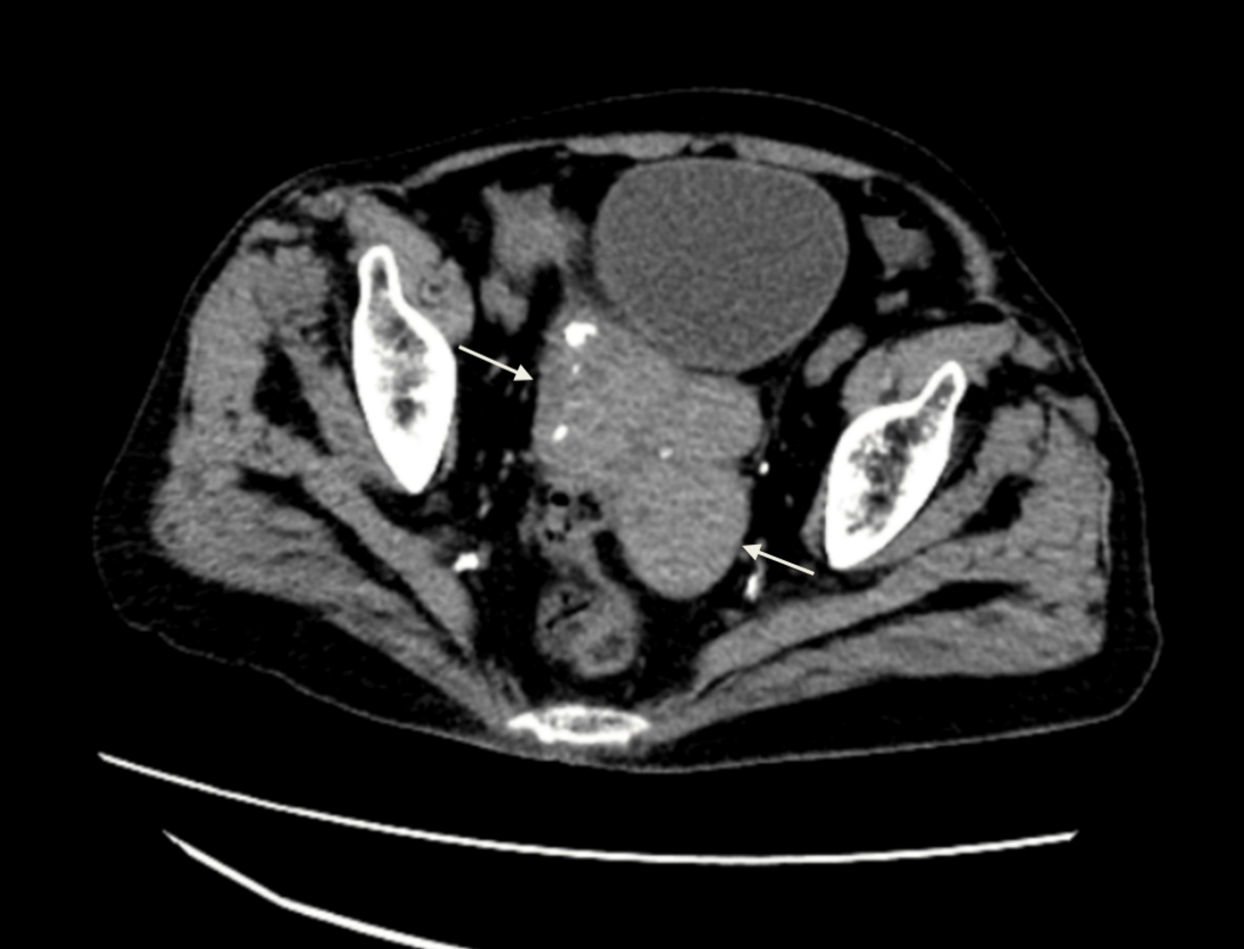
Axial view of the CT scan of the abdomen and pelvis shows a well-lobulated enhancing lesion of size 14.8 cm x 5 cm x 6.5 cm with a few small subcentimeter-sized foci of calcification noted arising from the terminal ileum bowel loops.

Computed tomography imaging also showed the presence of a lobulated mass lesion arising from the terminal ileum bowel loops (14.8 x 5 x 6.5 cm) with focal calcifications, without evidence of obstruction (Figure 3). Given the constellation of findings, the patient underwent exploratory laparotomy for further evaluation of the abdominal mass. 

**Figure 4:**
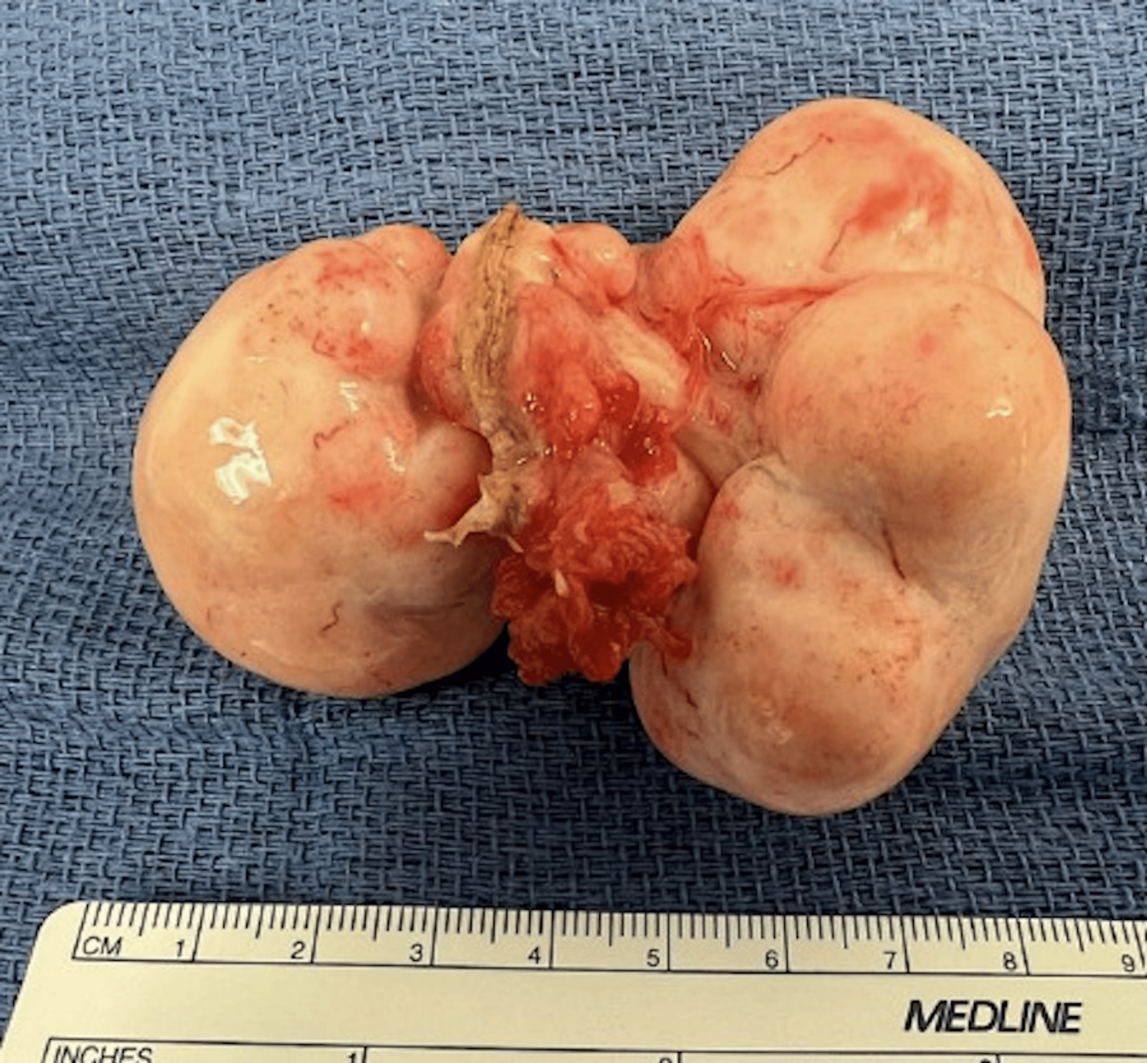
Gross specimen of right ovary consisting of a large rubbery mass overlying a small portion of the fallopian tube with fimbria; mass measures 8.2 x 5.5 x 4.2 cm. The mass outer surface is tan-white, nodular, and bosselated.

Upon entry into the peritoneal cavity, a large multilobulated ovarian mass was visualized in the right lower quadrant, measuring approximately 8 cm, accompanied by extensive retroperitoneal fullness (Figure 4). 

The mass was confirmed to be a leiomyoma and an 8.2 cm fibroma, necessitating oophorectomy and myomectomy. Subsequent pathology confirmed the presence of fibroma and leiomyoma in the uterus. The peritoneal wash fluid sample was negative for malignant cells, showing only rare mesothelial cells, few macrophages, and lymphocytes in a background of intact and hemolyzed red blood cells. CA-125 (8 U/mL) and CEA (<2.0 ng/mL) levels were within normal limits with a lactate dehydrogenase (LDH) of 306. The peripheral blood smear showed no abnormality.

Intraoperative findings were consistent with a diagnosis of Meigs syndrome, characterized by the presence of a benign ovarian fibroma (the 8.2 cm multilobulated mass on the right ovary) along with pleural and pericardial effusions.

Postoperatively, the patient experienced significant improvement in her shortness of breath, fatigue, and abdominal fullness. She recovered well and was discharged with appropriate follow-up plans. Given the findings of severe microcytic anemia, she was advised to undergo an endoscopy and colonoscopy to rule out any gastrointestinal pathology.

## Discussion

Our case report describes a unique presentation of Meigs syndrome in a 75-year-old African American female patient, highlighting several distinctive features and contributing to the expanding body of literature on this syndrome.

Meigs syndrome occurs in approximately 1% to 10% of ovarian tumors, with ovarian fibroma being the most frequent association [7, 8]. While instances have been documented in women under 30 years old, their prevalence is particularly higher in postmenopausal women like our patient, with the peak incidence observed in women in their seventies [7, 9]. Diagnosing Meigs syndrome requires a high index of suspicion, as it can mimic various other conditions such as ovarian malignancy, gastrointestinal stromal tumors (GISTs), leiomyomas, and metastatic lesions. In this case, the diagnosis was established through a combination of clinical evaluation, imaging studies, and the intraoperative findings of a large multilobulated ovarian mass, leiomyoma, and fibroma, in the absence of gastrointestinal pathology or liver metastases.

Serum CA-125, also referred to as cancer antigen-125 or mucin 16 (MUC16), is a glycoprotein biomarker found in the blood that is commonly used in clinical practice as a marker for ovarian cancer [7, 10]. It is produced by various tissues, including the epithelial cells of the ovary, and its levels can increase in response to various conditions, such as ovarian cancer, endometriosis, and Meigs syndrome. CA-125 levels may increase in severe congestive heart failure, menstrual cycle phases, and conditions like abdominal surgery, chronic obstructive pulmonary disease, active tuberculosis, and lupus erythematosus [9, 11]. However, its levels have been observed to be elevated in certain instances of Meigs' and pseudo-Meigs' syndrome.

In a systematic review examining CA-125 elevation in Meigs syndrome spanning from 1995 to 2022, a total of 36 patients with Meigs syndrome and elevated CA-125 levels were analyzed. The elevation of CA-125 varied widely, ranging from 42.3 IU/mL to 3969 IU/mL [9]. Notably, our patient's CA-125 level of 8 U/mL was within normal limits, contrasting with previous reports associating elevated CA-125 with Meigs syndrome [7, 12, 13]. It's crucial to recognize that CA-125 levels should not be solely relied upon to rule out malignant disease. Patients diagnosed with ovarian carcinoma often present with CA-125 levels within the normal range observed in healthy individuals before treatment.

The prevalence of pleural effusions in Meigs syndrome varies across studies but is estimated to occur in approximately 1% of cases [7, 10]. This prevalence can depend on factors such as the size of the ovarian fibroma, the presence of associated ascites, and the specific patient population being studied. In a single study, it was determined that only seven out of 447 patients (1.6%) with reported pleural effusion were able to be classified using Light's criteria [11]. Additionally, bilateral effusions as presented in our case are less common than unilateral effusions. A considerably higher number of patients exhibited pleural effusions on the right side compared to the left side or bilaterally (P < 0.001), with most of these effusions being exudative in nature [10, 11, 7]. Our patient’s pleural effusion resolved completely post-surgery, reinforcing the self-limiting nature of the effusions associated with Meigs syndrome.

Interestingly, our case also documented pericardial effusion, which is rarely seen in Meigs syndrome. Further comprehensive literature review reveals sparse documentation of Meigs syndrome cases exhibiting pleural and pericardial effusions concurrently. One such case involved an 84-year-old woman with a left ovarian fibroma who presented with dyspnea, pleural effusion, and pericardial effusion, ultimately diagnosed with Meigs’ syndrome following surgical resection of the ovarian tumor [5]. Similarly, another case reported a 55-year-old woman with a right ovarian tumor presenting with symptoms of cardiac tamponade, pericardial effusion, and mild bilateral pleural effusion, later diagnosed with a variant of Meigs syndrome termed Meigs-like syndrome [6].

The mechanism underlying the development of pericardial effusion in Meigs syndrome remains uncertain, as pericardial involvement in Meigs syndrome is exceptionally rare, but based on theoretical considerations and extrapolation from the known mechanisms of pleural effusion in MS, it is multifactorial. One proposed mechanism involves the secretion of vascular endothelial growth factor (VEGF) by the ovarian fibroma [12,13]. Vascular endothelial growth factor is implicated in peritoneal and pleural fluid accumulation in both malignant and nonmalignant diseases. In malignancies like ovarian cancer, VEGF promotes angiogenesis and vascular permeability, leading to ascites formation [13]. In non-malignant conditions such as Meigs syndrome, benign ovarian tumors produce VEGF, contributing to ascites and pleural effusion by increasing vascular permeability [12-14]. Additionally, inflammatory mediators released by the tumor or surrounding tissues may contribute to the formation of exudative effusions [15, 16]. Furthermore, obstruction of lymphatic drainage by the ovarian mass can result in impaired fluid resorption, exacerbating the accumulation of fluid in the pleural cavity.

The absence of ascites in our patient is also a distinguishing feature of her presentation. While ascites is commonly associated with Meigs syndrome, some patients present with atypical forms of the syndrome, showing little to no pleural effusion but significant ascites [16, 17]. The variability in clinical presentation underscores the importance of maintaining a high index of suspicion when evaluating patients with abdominal masses and effusions, as Meigs syndrome may not always manifest with the classical triad of ascites, pleural effusion, and ovarian fibroma. This case adds to the body of evidence highlighting the heterogeneity of Meigs syndrome presentations and reinforces the need for early and accurate diagnosis to prevent unnecessary interventions or misdiagnosis.

The prognosis of Meigs syndrome is generally favorable with prompt diagnosis and surgical management, as evidenced in our case, where the patient’s symptoms resolved post-operatively, including her shortness of breath and abdominal fullness. Surgical resection of the ovarian fibroma resulted in complete resolution of the pleural and pericardial effusions, supporting the role of surgery as the definitive treatment for Meigs syndrome [16, 18]. However, delayed diagnosis or inadequate treatment may lead to complications such as respiratory compromise, cardiac dysfunction, or progression to malignant disease [18]. Long-term follow-up remains essential to monitor for recurrence or the development of ovarian malignancy, although Meigs syndrome itself is considered benign.

This case highlights the complexity and variability of Meigs syndrome, particularly in patients with atypical presentations, such as the absence of ascites and the presence of bilateral pleural and pericardial effusions. The normal CA-125 level in this patient further emphasizes the importance of clinical judgment and comprehensive diagnostic workups. Given the rarity of Meigs syndrome and the diversity in its presentation, a larger case series would be invaluable to further elucidate the range of presentations, improve diagnostic accuracy, and refine treatment strategies for this enigmatic syndrome. Such studies would significantly contribute to our understanding and enable clinicians to sharpen their reasoning when managing cases of Meigs syndrome.

## Conclusions

This is a rare case report that sheds light on the diverse clinical presentations and diagnostic challenges encountered in Meigs syndrome. By documenting the rare occurrence of pericardial effusion alongside the more common pleural effusion and ascites, this case expands our understanding of the syndrome's spectrum of manifestations. Furthermore, the successful resolution of both effusions following surgical intervention underscores the importance of prompt diagnosis and appropriate management in achieving favorable outcomes. Further research and case studies are warranted to expand our understanding and refine treatment approaches for this rare syndrome.
